# Experience of Using an App in HIV Patients Older Than 60 Years: Pilot Program

**DOI:** 10.2196/mhealth.9904

**Published:** 2019-03-06

**Authors:** Julián Olalla, Jose María García de Lomas, Efrén Márquez, Francisco Jesús González, Alfonso Del Arco, Javier De La Torre, Jose Luis Prada, Francisca Cantudo, María Dolores Martín, Miriam Nieto, Javier Perez Stachowski, Javier García-Alegría

**Affiliations:** 1 Unidad de Medicina Interna Hospital Costa del Sol Marbella Spain; 2 Servicio de Farmacia Hospital Costa del Sol Marbella Spain; 3 Unidad de Investigación Hospital Costa del Sol Marbella Spain; 4 Asociación Concordia Antisida Marbella Spain

**Keywords:** HIV, aging, internet

## Abstract

**Background:**

New technologies can promote knowledge of HIV infection among patients suffering from this disease. Older patients with HIV infection represent an increasingly large group that could benefit from the use of specific apps.

**Objective:**

The aim of the study was to observe the acceptability and use of a mobile app on HIV infection in patients at least 60 years old and offer them the possibility of anonymously establishing contact with their peers.

**Methods:**

A series of clinical and psychosocial parameters were studied in 30 HIV-infected patients of over 60 years. The patients must be at least 60 years old, with a follow-up in the outpatient clinic for at least 1 year and without pathologies that limit his or her life expectancy to less than a year. They must know how to read and write. To be part of the group assigned to the app, they had to have their own smartphone and confirm that they were connected to the internet from that device. Overall, 15 of them were randomized to use an app and 15 were in the control group. All tests were repeated after 6 months.

**Results:**

The median age of patients was 66.5 years. Among them, 29 patients had an undetectable viral load at baseline. The median number of comorbid diseases was 2. Overall, 11 of them lived with their partners and 19 lived alone. They spent an average of 5 hours a day sitting down, and 56% (17/30) of them referred high physical activity. They scored 4 out of 5 for general quality of life perception. Moreover, 80% (24/30) presented high adherence to their treatment, and the average number of concomitant medications was 5. In the 6-min walking test, they covered a distance of 400 meters, and 3 of them desaturated during the test. The 15 patients made frequent use of the app, with 2407 sessions and an average of 7 min and 56 seconds time of use with a total of 13,143 screen views. During the 6 months of the trial, 3 non-AIDS events took place. There were no significant modifications to body mass index, blood pressure measurements, lipid profile, or immuno-virology information data. There were no differences in the questionnaire scores for perception of quality of life, confessed physical activity, or antiretroviral treatment (ART) and non-ART treatment adherence.

**Conclusions:**

Significant differences between studied parameters were not objectified in these patients, possibly because this trial has significant limitations, such as a small sample size and only a brief follow-up period. However, patients did use the app frequently, making this a possible intervention to be proposed in future subsequent studies.

## Introduction

### Background

The proportion of older adults infected with HIV grows progressively [1], not only because of increased survival of cohorts but also because of an increase in the number of new diagnoses in this group of population [2,3]. With the increasing age of our patients, the possibility of AIDS diagnosis event, serious non-AIDS events (osteoporosis, renal failure, vascular events, non-AIDS cancers, and neurocognitive impairment), or metabolic conditions (diabetes, hypertension, and hyperlipidemia) that are associated with increased morbidity and mortality rises [4,5]. All these conditions are encompassed in the concept of comorbidities. These patients are a population group in which the accumulation of different types of medications and the potential for toxicity and interactions will also be greater [6]. The social stigma that accompanies HIV infection is not negligible, especially in patients of older age; so being able to share their experience with their peers could lead to reducing feelings of loneliness and stigmatization. The use of these technologies in smartphones (mobile health) has also been developed in the HIV-infected population, although a specific HIV-infected older adults’ experience has not been reported yet [7,8]. Probably, compared with the younger infected ones, older adults are a differentiated group, who are less accustomed to the use of new technologies and have a special interest in being informed not only about HIV infection but also about the comorbidities associated with age. In addition, the isolation is greater in older individuals, so an app specifically designed for this group could be useful [9].

New technologies allow the creation of virtual communities that help to maintain the anonymity of the individual while allowing interaction with other peers [10]. The creation of peer support groups formed with reference and access to tips for health promotion and commented on by medical personnel news may have beneficial effects in this population. Anonymity could favor those people who would not participate in face-to-face activities or with fear of losing their privacy and those who could benefit from being supported by peers. This can be especially important in older people belonging to generations in which HIV infection or sexual condition was associated with a certain stigma in Spain [11].

### Objectives

The GoSHAPE 2015 program was supported by Gilead to promote the use of new technologies and the empowerment of patients. In this context, we deliver the development of an app. Being a pilot study, our objective was to observe the acceptability and use of a mobile app on HIV infection in patients at least 60 years old as well as offer the possibility of establishing anonymous contact between peers.

## Methods

### Investigative Team

The study was led by Costa del Sol Hospital Internal Medicine Unit (Marbella, Spain), in collaboration with the Pharmacy unit, the department of clinical investigation, and a local nongovernmental organization (Concordia). The approval of the local ethics committee was obtained, and all the patients signed informed consent before any study performance.

**Figure 1:**
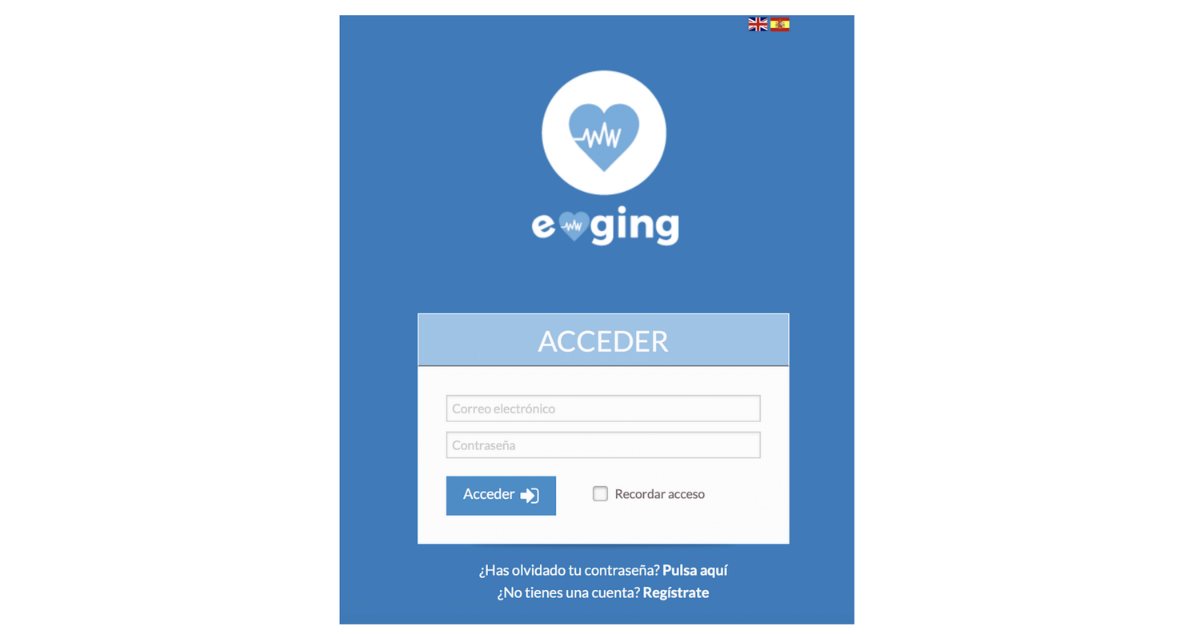
Interface of the app.

A prospective observational study was conducted in 2 phases. An app with different content was designed, and afterward, this app was tested with the patients over 6 months. The name of the app was e-ging (Figure 1). After registering, the patients accessed the menu screen, where they could choose among the different options of the app.

**Figure 2:**
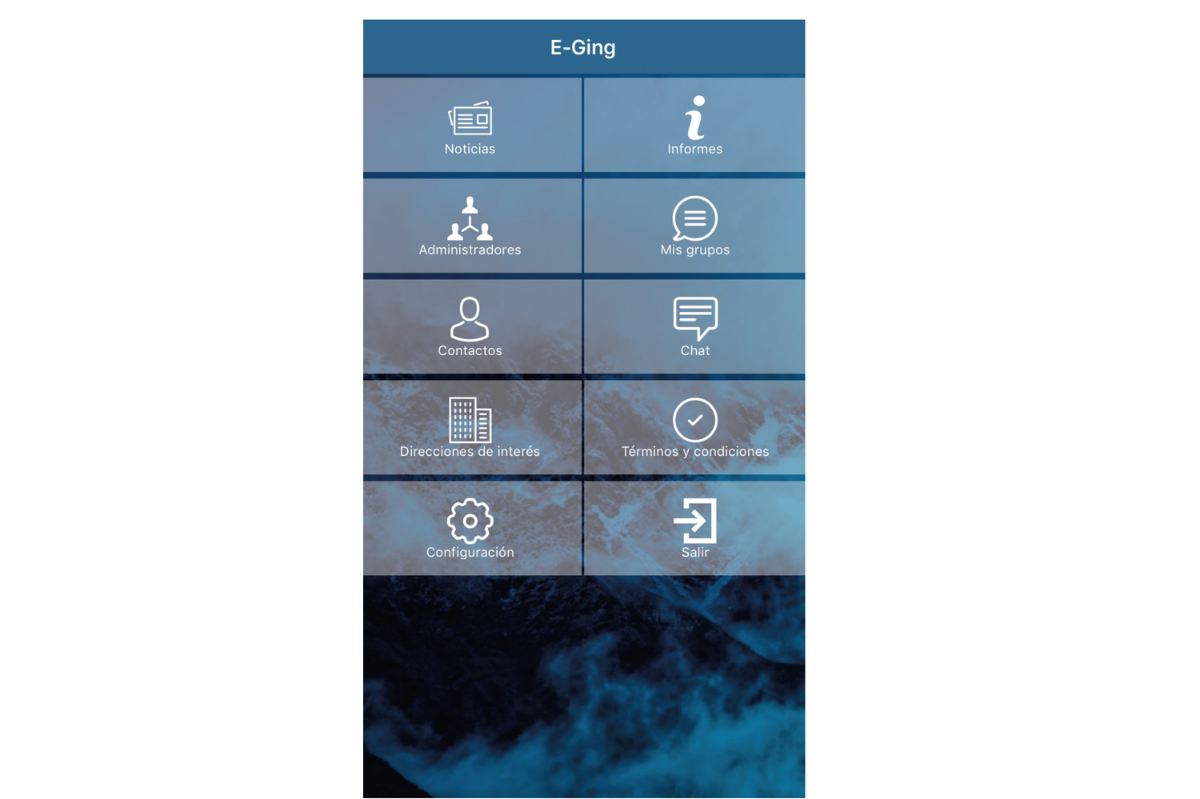
Menu screen.

The app consisted of 3 different sections, which can be seen in Figure 2:

- News (medical, health care, social, and healthy aging): Always focusing on commenting on news about HIV infection that was being spread in the national and international press. Some of the news was “What is osteoporosis and why is it produced?,” “alcohol causes 250,000 deaths from liver cancer per year,” “the dangers of high blood pressure,” “the epidemic of loneliness as a threat to health,” “the link between HIV and heart attack,” and “advances in the vaccine against HIV.”
- Reports: Information in understandable language about key aspects of the infection, such as the meaning of CD4 and viral load, antiretroviral treatment (ART) components, vaccine effectiveness, and smoking habit. Some of the reports were “What does CD4 and viral load mean?,” “What vaccines should I take?,” “What are opportunistic infections?,” “The importance of papilloma virus,” and “WHO recommendations on physical activity.”
- Chat: Designed for the purpose of anonymously exchanging opinions between patients. The patients were explicitly explained that the comments of the chat would be analyzed (in frequency, content, and number of participants) at the end of the study.

Both the news and the reports were uploaded by the research team, not by the patients, and both sections could be marked with *like* by the patients.

To have access to the app contents, the patients received a user number in a closed envelope and an access password assigned at random by computer software external to the project. Each user number had an avatar assigned to it as a profile to reinsure patient anonymity. To maintain privacy, the external aspect of the app and its name (e-ging) did not make any reference to HIV infection, and its use required a username and password each time the app was to be accessed.

#### Selection of Participants

Before recruiting the patients who collaborated in the study, a survey was conducted among all the patients aged at least 60 years in our HIV infection clinic, with a view to exploring their knowledge about the internet and new technologies [12]. Later, 30 of them were recruited. The patients must be at least 60 years old, with a follow-up in the outpatient clinic for at least 1 year and without pathologies that limit his or her life expectancy to less than a year. They must know how to read and write. To be part of the group assigned to the app, they had to have their own smartphone and refer that they were connected to the internet from that device. They were offered to enter the study according to their usual consultation. The patients who used the internet on their mobile phone (15) were offered to download the app, and those who did not have internet access on their mobile (the other 15) constituted a control group. There was no randomization.

#### Scheme of Visits and Information Collected

The app remained in operation for 6 months, after which it was canceled. During this time, clinical follow-up was the usual in both groups of patients (with or without app), with a visit to consultations every 4 to 6 months. No specific action was developed with the control group.

The following information was collected at baseline 6 months later:

1. Education level (referred by the patient himself).
2. Anthropometric information and blood pressure (taken in consultation, twice in recumbent position after 3-5 min of rest in that posture).
3. Blood count parameters: CD4 lymphocytes and viral load, lipid profile, and estimated glomerular filtrate using Chronic Kidney Disease Epidemiology Collaboration [13].
4. Comorbidities: cancer, diabetes, hypertension, dyslipidemia, osteoporosis or fractures, liver disease, cognitive deterioration, and tobacco consumption (for all these: yes or no). This information was taken from the patients’ medical records, if the patients had recorded this diagnosis between the clinical judgments.
5. Medication: type of ART received and chronic non-ART medication.
6. Adherence to ART and non-ART medication: calculation using dispensation of ART registries provided by the pharmacy unit, followed by the Simplified Medication Adherence Questionnaire (SMAQ) [14].
7. Adherence to chronic non-ART medication: the Morisky-Green-Levine questionnaire [15].
8. WHOQOL-BREF, World Health Organization Quality of Life Questionnaire (short version): it evaluates physical and Psychological quality of life, social relationships and satisfaction with health care and social services [16].
9. International Physical Activity Questionnaire (IPAQ): classifies weekly physical activity as high, moderate or low [17].
10. 6-min walking test (6MWT): reflects the physical capacity of an individual to perform the physical activities of daily life.

In addition to these questionnaires, patients were asked to rate their quality of life perception ranging from 1 (worst) to 5 (best), and the same for the perception of health.

Analytical parameters and 6MWT results were collected to explore if a short intervention could improve physical, functional, and analytical status of our patients.

#### Data Analysis

For the analysis of the chat at the end of the experience, a dictionary of words and terms of interest was put together to be able to discard irrelevant words for the purpose of the project. The choice of relevant terms was agreed to by the research team. Once the dictionary was available, an algorithm was implemented that searched for coincidences in all chat messages. The terms were grouped into areas for this purpose, and in these areas, words were classified as *key words*, which is the term or expression that we want to quantify, or its *variants*, which are variations of that word or expression. With this structure, a search was implemented in the whole chat. The areas into which the key words have been grouped are as follows: pathologies, medications, nutrition, healthy attitudes, sanitary and professional centers, psychological, and emotional. In the pathologies area, a second analysis of key words was made and grouped into the following areas: cardiovascular disease, cancer, HIV, and osteoporosis. The contents of the chat were analyzed by 3 members of the research group so that the comments were framed in the context of these key words.

After closing the app, the possibility was offered to send a brief written opinion about the experience of using the app to the 15 individuals who had downloaded it.

Quantitative data are provided as medians with an interquartile range, and qualitative information is given as percentages. A nonparametric procedure (Mann-Whitney U) was used to compare quantitative variables, and proportions were compared using the chi-square test. IBM SSPS 20 was used for statistical analysis.

## Results

### Population Characteristics

Baseline characteristics of the patients are shown in Table 1. All of them were receiving ART.

As for physical activity measured according to the IPAQ, 2/30 patients (7%) referred low activity, 11/30 (37%) moderate, and 17/30 (57%) high. They stated that they spent 5 hours sitting down (0-12). The quality of life perception was 4 (2-5). With that same scale, the perception of health was 3 (2-5).

The number of concomitant medications was 5 (0-12), and 17/30 (57%) patients were using at least four medications.

There were no significant differences found between the group that downloaded the app and the control group in any of the variables mentioned.

Over the 6 months of this project, there was no registry of any AIDS events, but there were 3 non-AIDS events: one sudden death in the group not assigned to use the app and 1 heart attack and colon cancer in 2 patients in the app use–assigned group.

There was no significant modification in body mass index parameters, blood pressure, lipid profile, or immuno-virology information data. There was no significant modification of the distance covered in the 6MWT. There was no significant modification in the perception of quality of life questionnaire scores, confessed physical activity, or ART or non-ART adherence. Table 2 shows the main analytical parameters, adherence to treatment, quality of life, and the 6MWT results at baseline and 6 months, without any significant differences being observed in any case.

### Use of the App

The app was in use between April 30, 2017, and October 31, 2017. During these 6 months, 19 reports and 75 news were uploaded. There was a total of 2407 sessions and an average session time of 7 min and 56 seconds. The screen views ascended to 13,143, with an average of 5.46 screens per session.

The most visualized screens were the *Chat* (4046 views) and *News* (890) screens, at a considerable distance behind them were *Contacts* (96), *Reports* (90), and *Useful addresses* (66).

**Table 1:** Baseline characteristics of the patients (N=30).

Demographic characteristics		Statistics
Age in years, median (range)		65.5 (60-78)
Female, n (%)		5 (17)
**HIV infection, median (range)**		
	Years since diagnosis	11 (2-31)
	Years since beginning of ART^a^	10.5 (2-28)
**Way of transmission, n (%)**		
	Men who have sex with men	11 (37)
	Heterosexual	17 (57)
	Former injection drug user	1 (3)
	Unknown	1 (3)
CD4 lymphocyte nadir (cell/µL), median (range)		195 (4-851)
Number of lines of ART during life, median (range)		4 (1-11)
**Comorbidities**		
	Number of comorbidities, median (range)	2 (0-6)
	Dyslipidemia, n (%)	18 (60)
	Hypertension, n (%)	14 (47)
	Diabetes, n (%)	11 (37)
	Chronic liver disease, n (%)	9 (30)
	Active tobacco consumption, n (%)	11 (37)
**Cancer, n (%)**		
	Former	4 (13)
	Active	1 (3)

^a^ART: antiretroviral treatment.

### News and Reports

There were 75 news publications in different categories: HIV (34 published), healthy aging (27), project information (7), social and psychological (5), and Information and Communication Technologies (ICT) use and the elderly (2). A total of 135 likes were received, the most liked being “On going” (5 likes), “How walking a dog can help the elderly comply with physical exercise recommendations” (5), “How to get brown in a healthy way” (5), “Welcome!” (4), “A cure for HIV?” (4), “Food for people with HIV” (4), “Is the flu vaccine less effective in overweight people” (4), “A monthly injection can maintain AIDS virus confined” (4), and “Spain fails in recommended calcium intake” (4). There were 22 news comments, the most commented paper being “Around 5000 patients have HIV infection in Malaga province” (3 comments).

### Chat Analysis

As an example, in the section corresponding to *psychological*, the key words were happiness, emotion, sadness, surprise, restlessness, and greetings. Within the key word *sadness* were included chat comments such as “you lose the desire of everything,” “today I’ve been very dejected and sad,” “we all have a bad day,” “I’m pulling, I have head like a pot of crickets,” “I never dream,” “I’m fed up,” “I do not know anything about anyone,” “I’m worried, without knowing anything,” “this heat kills me,” “hello guys you’re going to forgive me I’m not for nothing,” “it was a horrible time and without being able to talk to anyone, it passes very badly,” “I have the body as if I had been beaten,” “no one knows what it is,” “absolutely nobody in my family knows anything,” “I’m bored,” “my friends do not know after twelve years,” “that’s why it was so hard for me to admit it, it cost us much assimilate, fuck has cost me,” “I do not understand how people are so denied,” and “what a pity, we are the only survivors.”

**Table 2:** Evolution of the main parameters evaluated.

Parameter		Baseline	6 months	*P* value
**Analytical parameters**				
	CD4 lymphocyte (cell/µL), median (range)	723 (166-1251)	811 (178-1833)	.08
	HIV-1 viral load <50 copies/mL, n (%)	29 (96)	28 (93)	.44
	Total cholesterol, median (range)	190 (106-306)	178 (106-285)	.77
	HDL^a^-cholesterol, median (range)	49 (21-81)	47 (23-94)	.24
	LDL^b^-cholesterol, median (range)	116 (37-190)	108 (44-195)	.95
	Triglycerides, median (range)	115 (28-519)	118 (50-274)	.19
**Adherence, n (%)**				
	ART^c^ adherence >95%	26 (87)	27 (88)	.56
	ART adherent (Simplified Medication Adherence Questionnaire)	24 (80)	22 (73)	.66
	Non-ART adherent	20 (66)	17 (58)	.49
**Questionnaires**				
	WHOQOL-BREF Physical quality of life (score)	69	69	.13
	WHOQOL-BREF Psychological quality of life (score)	75	78	.19
	WHOQOL-BREF Social relationships (score)	69	69	.32
	WHOQOL-BREF Satisfaction with health care and social services (score)	75	69	.13
	IPAQ^e^: moderate-high activity, n (%)	28 (93)	27 (92)	.16
	Perceived QOL^f^ (score)	4	4	.22
	Perceived health status (score)	3	4	.19
	6-min walking test (meters)	400	410	.76

^a^HDL: High density lipoprotein.

^b^LDL: Low density lipoprotein.

^c^ART: Antiretroviral Treatment.

^d^WHOQOL-BREF: World Health Organization Quality of Life Questionnaire (short version).

^e^IPAQ: International Physical Activity Questionnaire.

^f^QOL: Quality of life.

As we already stated, terms were grouped by categories, where we can find the following:

- Nutrition: terms related to the nutrition field were mentioned 104 times, where *coffee* was the most frequently mentioned term.
- Health care environment (sanitary and professional centers): terms related to health care environment were mentioned 187 times, with *doctor* being the most named term (124).
- Healthy attitudes: terms related to healthy attitudes were mentioned 273 times, the most mentioned terms were *beach* (94) and *walking* (87).
- Medications: terms related to medications were mentioned 115 times, the most named terms were medication or treatment (28) and *acenocoumarol* (24).
- Pathology or disease: terms related to pathology or disease were mentioned 414 times, especially *weight or height* (76), *blood pressure* (76), cancer (24), *heart or heart attack* (21), *depression* (20), and *diabetes* (18).
- HIV: was quoted 184 times, with references to health care environment above all (*doctors*, *internal medicine outpatient consultation room*, *Costa del Sol Hospital*, and *in good hands*) being 157 and only 9 direct references to HIV.
- Psychology and emotional: terms related to psychology and emotional were mentioned 554 times, the most used terms (or similar) being *greetings* (305), *happiness* (135), *emotion* (70), *anxiety or mood* (27), *surprise or wishes* (11), and *sadness ormelancholy* (6).

The chat participants described the experience as purely positive; it allowed them to create “a space for freedom in which they could talk to other elderly patients infected,” they found “nice people with my same problems,” and all of them would repeat the experience. All the patients that downloaded the app referred that their knowledge about the infection had increased and their feelings of *secret* or *taboo* toward the disease had lessened.

## Discussion

### Principal Findings

In our experience, the use of a mobile app in older adults infected with HIV has translated into a frequent use of this app, with just over 2400 sessions and an average time of use of almost 8 min for each one of them. Far from being a collective that shies away from mobile app use, it is quite clear that if this population’s interest is stimulated toward these apps, they will make use of them and make the most of the information they have to offer. The fact that the navigation in each session implies an average of 5 screens in each one of them also shows user’s interest for the different sections of the app and that navigation is certainly dynamic. The most frequently viewed screens were *Chat* (over 4000 times) and *News* (over 800 times), which reveals that patients were clearly interested in the social relationship that the app implied and in commenting on latest medical news.

The population of our study responds to a pattern of comorbid and polymedicated patients (a median of 5 medications excluding antiretroviral), diagnosed at around 50 years of age and with a decade of ART use. Although the perception of their health was 3 over 5, the perception of quality of life was higher (4 out of 5), which definitely shows certain optimism about their own life in spite of the illness, verified again in the WHOQOL-BREF results, where the psychological quality of life results were higher than the physical quality of life ones. The high appreciation of health care and social services also stands out (75 over 100).

The use of the app was not associated with clinical or analytical parameter changes in patients. Changes in this type of parameters are probably not to be expected in this type of elderly population in such a short period as 6 months and also because of the low number of patients included in each group (n=15) of this pilot study.

Analysis of the chat reveals that the main area of concern for patients is disease in general, a lot more than HIV, referring to typically age-related pathologies rather than the chronic infection itself. The analysis of feeling-related terms reveals a positive attitude, with frequent mention of happiness.

### Strengths and Limitations

Compared with other studies [18], our study population had physically active perception of itself (over 50% referred high physical activity), which could also be related to a better perception of their psychological welfare. Indirectly related information that supports this fact is that *beach* (94) and *walking* (87) stand out among the most quoted individual terms in the chat.

Experiences of the development of apps in HIV have been published with the purpose of improving adherence [19], but perhaps, elderly HIV-infected patients represent a group in which this type of apps can promote stigma reduction and sharing experience between peers. The use of mobile apps, where anonymity is preserved, can help reduce the social barriers that these patients have to confront [20]. The presence of this stigma can be associated with worse ART adherence rates and less viral suppression rates [21-23]. However, in our patients, adherence to ART was very high, although it could be improved in non-ART medication. Comorbidity presence related to age translates into a higher use of these medications, and making this treatment easier (lower amounts of pills, and precise indications) must be another step in the reinforcement of this adherence.

Similar to other mobile phone–based interventions [24], ours has not demonstrated a significant increase in ART adherence, although the truth is that the initial situation was very positive in that parameter (almost 90% referred over 95% adherence).

### Conclusions

Ours constitutes a preliminary experience that seems to confirm that our older adults with HIV infection find the utilization of a mobile app to be able to receive information about the infection and connect with individuals with their same problems to be useful. Studies larger than ours, and probably more extended in time, should confirm if some kind of benefit is obtained in this group of patients (older adults with HIV infection) through new technologies.

## Abbreviations

ART: antiretroviral treatment
IPAQ: International Physical Activity Questionnaire
ICT: Information and Communication Technology
6MWT: 6-min walking test
WHOQOL-BREF: World Health Organization Quality of Life Questionnaire (short version)
